# Preparing for and preventing dengue explosion in East Asia after border reopening

**DOI:** 10.1016/j.lanwpc.2021.100378

**Published:** 2022-01-15

**Authors:** Hsiang-Yu Yuan

**Affiliations:** a Department of Biomedical Sciences, Jockey Club College of Veterinary Medicine and Life Sciences, City University of Hong Kong, Hong Kong SAR, China; b Centre for Applied One Health Research and Policy Advice, Jockey Club College of Veterinary Medicine and Life Sciences, City University of Hong Kong, Hong Kong SAR, China; c Guy Carpenter Asia-Pacific Climate Impact Centre, City University of Hong Kong, Hong Kong SAR, China

The spread of many infectious diseases, including dengue fever, has been largely affected by Non-Pharmaceutical Interventions (NPIs) in response to the COVID-19 pandemic.

Dengue is one of the most rapidly spreading mosquito-borne viral diseases in the world and has frequently caused outbreaks in tropical regions. In general, high humidity and warm temperature are conditions that favour mosquito breeding and reproduction. Thus, climate change (such as increased temperature and rainfall and extreme weather events) may facilitate dengue expansion.^1^ In East Asia, climate change has raised concerns that dengue fever has become endemic, particularly in cities or countries neighbouring the Tropic of Cancer.^2^

Dengue outbreaks in subtropical regions in East Asia, such as southern provinces in China, Hong Kong and southern Taiwan, were commonly thought to be caused by imported cases during summer. However, recent outbreaks in these areas are of a great concern because of worry about the endemicity. For example, in 2014 and 2015, historical high records in Dengue outbreaks occurred in Taiwan,^3^ and Guangdong, China.^4^ Hong Kong also faced a highest number of local cases later in 2018.^5^ A key question is whether these regions still remained non-endemic.

Furthermore, knowing whether international travel restriction is able to reduce the outbreak is critically important for seasonal epidemic regions. Lockdowns and border restrictions implemented in 2020 provide an opportunity to look at their impacts. With more people spending more time at home, lockdowns are likely to influence the contact between human and mosquito populations.^6^ Many endemic countries have reported worse than average dengue incidence after lockdowns or travel restrictions were implemented,^7^ presumably staying at home longer may increase the transmission of dengue. On the other hand, whether the international travel restriction can reduce the dengue incidence in places with seasonal dengue outbreak is still unknown.

Yunnan, a province of China, located within 10°C January isotherm and neighbouring the Tropic of Cancer, is at high risk of transforming into endemic under the influence of global warming. High regional synchrony in dengue incidence was observed between Yunnan and surrounding countries, such as Laos, Thailand, and Vietnam, all belonging to endemic regions. Li and co-authors used an integrated approach (phylogeographic and statistical analyses) to confirm that Yunnan remained a non-endemic area by comparing dengue data before and after the beginning of COVID-19 pandemic.^8^ Phylogeographic analyses were performed using BEAST to reconstruct the spatial dynamics of dengue virus (DENV) 1-4 from sequences containing the location traits, and to infer ancestral branch locations.^9^ For each serotype, support for non-zero rates of exchange between all pairs of locations was evaluated using Bayesian stochastic search variable selection (BSSVS). Transmission history was inferred from the Markov jump counts, the total number of transitions between the nodes in phylogenetic trees. The exported and imported events can thus be estimated by calculating the total number of jumps for different regions.

In the year 2020, the number of dengue cases reduced significantly. Li et al. further showed that the impact of travel restriction is significant on dengue cases using a quasipoisson regression model taking account of weather predictors (i.e. temperature and rainfall).^8^ The results confirmed that dengue incidence between 2013-2019 in Yunnan was closely linked with international importation of cases from neighbouring countries and the reduction in 2020 was largely due to travel restrictions. These results along with phylogeographic analyses suggest that Yunnan is a regional sink for DENV lineage movement and that border restrictions may have substantially reduced the burden of dengue.

Preparing for and preventing a larger dengue outbreak after reopening international borders in these subtropical regions becomes urgently important. Improving dengue mosquito surveillance and testing and tracing policies for travellers arriving from endemic countries during large outbreaks or high risk dengue seasons may become critical steps to predict and control the global spread of dengue.

## Declaration of interests

Author has no conflicts of interest to declare.
